# Summary of Progress in the Medical Sciences

**Published:** 1875-09

**Authors:** 


### Summary of Progress in the Medical Sciences.

### I.—Obstetrics.

1. *Diagnosis and Treatment of Placenta Prævia.* Charpentier. (*Archives de Tocologie.*)

(*A.*) Date of expectation: from seventh to eighth month, rarely the sixth. *Sudden* occurrence and cessation of hæmorrhage, with reappearance at uncertain intervals, and in increasing quantities, till labor supervenes. Absence of ballottement, from interposition of placental mass. Hæmorrhage always external, and continuous with uterine diastole. Expulsion during systole. (*B.*) Artificial delivery most dangerous, and only suitable to urgent cases. Rupture of membranes only admissible when there is partial dilatation—difficult in complete presentation. Ergot greatly increases danger to child, and is contra-indicated in cases of pelvic contraction, organic uterine disease, and mal-presentation. The author regards the plug of charpie or tow (a pound and a half of the former) as best treatment, bladder and rectum having been previously emptied.

The charpie is introduced in the form of small balls, well covered with cerate, twenty or thirty of which have a thread attached. The anterior and (especially) the posterior culs-de-sac are tightly packed, the vagina being filled till the pellets appear externally. Then a handful or more of dry charpie is applied, covered with three or four compresses, and fixed by a T bandage, the whole being left in position from twelve to twenty-four hours. If the charpie at the vulva becomes moist, the plug is imperfect, and should be reapplied.

2. *Sulphate of Quinia as an Abortifacient and Oxytocic.* Chiari. (*Gazetta della Cliniche*, No. 29.)

The author concludes that the disulphate is not an abortifacient, and will not certainly induce premature labor, with or without the aid of mechanical measures.

It should not be relied upon in treating slow, suspended, or irregular labor ; and, when indicated by morbid symptoms during pregnancy, it should be administered, not only for relief of the latter, but to prevent abortion or premature labor. The remedy has no modifying influence upon puerperal affections of infectious or sporadic origin.

3. *Treatment of Uterine Spasm.* FRAENKEL. (*La France Medicale*, June 12, 1875.)

The author recommends a novel treatment for relief of uterine spasm during the expulsion of fœtus and placenta. When the spasmodic contraction cccurs, whether it affects the whole or a part only of the uterus, whether it acts upon the fœtus or only the placenta, and when introduction of the hand is impossible, it is proposed to give a sub-cutaneous injection of a milligramme of the sulphate of atropine, and of fifteen milligrammes of hydro-chlorate of morphia in solution. Chloroform is then to be inhaled for five minutes. Resolution of spasm rapidly follows, and delivery is not followed by hæmorrhage.

4. *Extra-Uterine Pregnancy.* CONRAD. (*Correspondenz Blatt fur Schweizer Aertze*, No. 5.)

Three cases are reported. In the first, a primipara, an abdominal enlargement was found to be occasioned by left tubal pregnancy, accompanied by grave symptoms. The fœtus formed an encysted tumor, which could be detected through the abdominal walls. An abdominal incision was made, about the middle of the fifth month, and, by that means, bones of the cranium, with other bones and diaphyses, ribs and debris of membrane, were removed. At the end of the sixth month, the resulting fistula closed, and the patient was well—a small painless tumor remaining on the left side of the uterus.

In the second case, pregnancy was complicated by the discovery of a species of mole—the fœtus giving evidence of calcification. Here, also, the patient survived.

In the third case, there were symptoms of peritonitis about the third month. While the possibility of extra-uterine pregnancy was admitted, there was still some doubt as to whether a diagnosis should not be made between pelvi-peritonitis and retro-uterine hæmatocele. Rectal puncture, a few inches above the sphincter ani, gave exit to some clots, and more than an ounce of liquid blood. The patient died in collapse, eight days afterward, and, upon post-mortem examination, the bones of a three months' fœtus were discovered in a sac formed of plastic lymph, to which the intestinal loops adjacent had become agglutinated.

5. *Extra-Uterine Pregnancy.* Bandl. (*Wiener Medizinische Wochenschrift,* No. 32.)

The author reports a case of a woman, 35 years of age, having two distinct abdominal tumors. The left, which was the larger, contained a living child. That upon the right side was of a doubtful nature. The uterus was empty. During the third month of pregnancy, the patient had suffered from symptoms which pointed to the passage of a fœtus into the abdominal cavity. Objection was made to operative interference, and the patient died in violent pain, with great increase in bodily temperature.

Five minutes after death, Dr. Bandl removed a fœtus weighing about nine pounds, which died of asphyxia after the expiration of ten minutes. The cavity in which it had rested was formed, anteriorly and posteriorly, by the abdominal parietes, covered with a dense false membrane, and by agglutinated loops of the small intestine, and the ascending and descending colon. The floor was formed of the pelvic viscera equally adherent to each other. The uterus was five inches in length, and pushed over to the left side. The tumor which had been discovered on the right side, contained the placenta. Near the latter was found the umbilical cord, surrounded by a brown and yellowish envelope, made up of retracted membranes.

### II.—Gynecology.

1. *The Epithelium of the Mammary Ducts.* Robin, Ch. (*Jour. de L'Anatomie et de la Physiol.*, No. 4.)

The galactophorous canals are not provided with a mucous lining. Their epithelium is made up of a thick layer of many-sided, almost prismatic cells, having a tolerably large and distinct nucleus. It rests upon a structureless limitary membrane, from one to two-tenths of a millimetre in thickness, whose adherent surface becomes insensibly united with the laminated tissue of the mammary structures beneath. Ordinarily, there are two rows of epithelial cells in this lining membrane, the deepest composed of nucleated epithelium (or "recently-individualized cells") only one-hundredth of a millimetre in thickness, having an oval nucleus and no nucleolus. The nucleolus is, however, to be discovered in the nucleus of the epithelium of a certain number of subjects in a healthy condition. In almost all cases of tumors of the breast (the so-called cancerous neoplasms and others), the galactophorous canals which are not destroyed, exhibit a distinctly marked pavement epithelium, composed of fine cells from two to three times larger than those ordinarily described, more or less covered with fatty granulations, and exhibiting a distinct oval nucleolated nucleus, larger than in the normal condition.

2. *The Development of the Ovary.* De Sinety. (*Le Progres Medical*, June 19, 1875.)

After the third month of intra-uterine life, two species of cells can be discovered in the ovary. Some are quite small, others are larger. Those having a large nucleus are primordial ovules. They are disposed in an irregular order, and form minute masses, separated from the connective tissue by delicate layers. At this time—as at the moment of birth—the proper ovary is merely represented by that which eventually becomes the cortical substance.

The medullary substance is represented merely by embryonic connective tissue, in the form of a small pedicle, and the vessels which are supplied to the ovary. At the fifth month, primordial follicles can already be discovered, made up of a layer of connective tissue and a circlet of small cells about the ovule. At the seventh month, the primordial follicles become more numerous, and the so-called "canals of Pflüger" appear, which are changed into follicles by a species of strangulation.

But about the ninth month, and during the few weeks succeeding birth, these canals anastomose, communicate with the epithelium of the ovarian surface, and contain the cells which characterize that epithelium. In the ovaries of the newly born, Graafian follicles are almost always visible to the naked eye, contrary to the generally received opinion on this point.

In the few days which succeed birth, follicles may often be discovered, as well developed as in the adult female, and constituting true cystic ovaries, in which are to be seen ovules whose origin is indubitable. This fact is probably intimately connected with certain mammary phenomena, noticeable at the same age. Observations upon the testicle, at this period, would undoubtedly prove interesting, as tending to throw light upon the puberal changes in both sexes.

In the cystic ovaries of infants, there are often cicatrices and follicles in different stages of atrophy—a circumstance which points to the possible resorption of follicles, in spite of the considerable development which they have attained. These cicatrices are important when considered with reference to the ulterior development of the ovary. During the process of their organization, the vascular and connective ovarian tissues penetrate deeply beneath ; and thus the medullary substance is formed, which pushes more and more to the periphery the ovule-bearing cortical layer.

At the fifth year, the medullary and cortical portions are equally represented in the substance of the ovary, while in the adult the cortical portion does not represent more than a quarter of the entire organ. At any period of life, the Graafian follicles which are most developed are always found in close proximity to the vascular portion. Perhaps the activity they display is due to the excess of nutriment which they thus receive.

3. *Ovariatomy Complicating Pregnancy.* HILLAS, THOS., M.R.C.S. (*Australian Med. Jour.*, Feb., 1875.)

June 13, a single woman, 24 years of age, was placed under chloroform, and an incision made midway between the umbilicus and pubis, in commencing ovariotomy. When a small wound was inflicted upon the peritoneum, a large jet of venous blood escaped, and was controlled by pressure. The operator was convinced that he had wounded a gravid uterus, and extended his incision to the umbilicus, when a large uterus rolled out upon the thighs, and the ovarian sac protruded. Eleven quarts of fluid were drawn off by tapping; there was no hæmorrhage, and but few adhesions. The sac contained a dozen small cysts. The short and thick pedicle was finally tied firmly with double whip-cord ligature. Afterward, the clamp was applied, the pedicle divided, and the ends of the double ligature tied over the clamp. The uterus was then incised to five inches, and the placenta, with a well-developed eight-months fœtus, extracted—the latter living. The uterine wound was closed with silver wire sutures, when the organ properly contracted. In six weeks the cure was complete. She has since menstruated regularly.

### III. CUTANEOUS AND SYPHILITIC DISEASES.

1. *Visceral Syphilis.* COFFER. (*Le Prog. Medical,* June 19.)

A woman, 32 years of age, died at the Lariboisiere, having exhibited ulcers of tubercular syphilis upon the legs, osteo-periostitis of one malleolus, hæmatemesis, double hydrothorax, anasarca and hepatic enlargement.

At the autopsy, the two inferior lobes of the lungs were found affected with interstitial pneumonia, and, at one point, there was considerable thickening of the connective tissue, with bronchial dilatation. The liver was voluminous and completely deformed by retraction of a largely-developed connective tissue. Its surface was deeply furrowed and covered by projections and broad fibrinous bands. On section, a white resisting fibrous mass was found, penetrating deeply into the hepatic tissue, having in its centre, small spherical, yellowish, indurated masses of a caseous appearance, each as large as a pea.

The microscope indicated that the newly developed connective tissue, not only surrounded the lobules but *penetrated into their interior.* Amyloid and fatty degeneration of the hepatic cells coexisted.

The case was regarded as one of visceral syphilis, with production of gummata in the lungs and liver. In the discussion upon this case, M. Després, of the Academy of Medicine, objected to the diagnosis, since the history of antecedent syphilis was wanting. It was not clear to him that the ulcerations upon the leg were syphilitic and with the naked eye he could detect merely the evidences of hepatic cirrhosis.

2. *Anal Eczema.* VÉRITÉ. (*La France Medicale*, June 26, and July 3.)

In this affection vesicles are rarely observed on account of the early resorption of their contents and their change into papules. When ruptured, the concretion of their contents produces delicate yellow or greyish scabs.

There is generally a red or rosy surface about the anal orifice, covered with liçhenoid papules, having a scratched summit. According to the state of the sweat glands, more or less numerous and fine crusts are to be seen. The cutaneous folds in the neighborhood of the anus, may be œdematous, fissured, or exhibit ulcers extending as far as the derma. The perineum, scrotum, sacral region, vulva, and internal surface of the thighs, may be invaded.

The moisture is due to glandular secretion—not to eczematous discharge. Anal eczema is a dry eczema in a humid locality. According to Bazin it is preceded by pruritus, and its excessive duration is due to the presence of sweat and fœcal matter.

For relief of the pruritus, patients will press aside the nates to get at the mucous surface; and not only rub and scratch it, but introduce two or three fingers into the rectum for several minutes, thus producing a titillation of the mucous surface which is followed by a slight serous discharge, succeeded by relief and sleep. This habit, difficult of renunciation, is dangerous in proportion as it is accompanied by pollution.

The consequent changes are analogous to those described in legal medicine as characteristic of passive pederasty: an infundibulum is formed—fissures and crests ensue; the semi-spherical concave is transformed into a plane which extends from one ischiatic projection to another—all of traumatic origin.

In women there is often vulvar pruritus, erythema of the labia majora and epithelial desquamation of the cervix. In both sexes there may be hæmorrhoidal complications. When the venous circulation is interrupted, there is infiltration and thickening, excessive pigmentation, even to the production of a bluish tint, irregular reproduction of the epidermis, and dermatitis, but no eczema proper, since the serosity escapes from the hyperemic corium.

The treatment should consist of alkalies internally, with topical application of ferric sulphate. Hardy recommends glycerine in which is dissolved a small quantity of the nitrate of mercury, when the pruritus is excessive. Other preparations recommended are corrosive sublimate, white precipitate, and solutions of potassium bromide.

The presence of worms is a frequent exciting cause of this disease, and calls for vermifuges, which, however, do not always relieve the eczema. The hypochondriasis and singular conceptions of some patients respecting this malady, are to be considered in the prognosis. Spontaneous cure occasionally occurs. Cancerous degeneration of the rectum rarely complicates these cases.

3. *The Nervous System and Skin Diseases.* BULKLEY. (*Archives of Electrol. and Neurol.*, May, 1875.)

There are two important causes of skin diseases: 1, Reflex nerve irritation; 2, Direct nerve irritation. The first is shown *inversely* in the occurrence of intestinal ulceration attending extensive cutaneous burns: *directly*, in hot, pale, perspiring, or flushing surfaces, where these changes are induced by ingesta and mental or nervous states, *e.g.*, pruritus and urticaria, also herpes gestationis, which is relieved by emptying the uterus. The second cause produces such lesions as those of zoster, leprosy and syphilis; and it is effective in patients troubled with gout, mal-assimilation, oxaluria, lithæmia, etc. To this class belong the rashes produced *some time after* ingestion of fruits, fish and the rash-producing medicaments.

Tar and buckwheat will produce acne; but so will an ulcerated uterus—the former, possibly, by distributing an irritating substance through the vascular system, the latter by reflection of irritation to the skin. If inflammation of spinal ganglia will produce the bullæ of zoster, excessive heat or cantharides externally, as well as the iodide of potassium internally, can produce similar effects. As to pemphigus, it may be syphilitic; may it not be in some instances a neurosis?

In lithæmia and icterus, the circulation of effete products produces pruritus—but so may flea-bites, the canaliculus of the acarus, the impregnated or diseased uterus, the ascaris in the rectum. Lepra anæsthesia is, more incontestibly than all others, a nervous disease of the skin, but its phenomona are often those of syphilis, and the lesions of the latter disease may be ultimately occasioned by alterations in the structure or function of nerves. Until more is known respecting the pathological changes in nerves and ganglia supplying diseased skin, we are hardly prepared to isolate a few cutaneous disorders, with more obvious nerve-phenomena, and call them neuroses.

### IV. Surgery, Ophthalmology and Otology.

1. *Amputation through joints.*

In amputations through joints for accidents, Dr. Geo. Macleod (*Glasgow Med. Journal*, Oct., 1874,) obtained very good results by not interfering with the cartilage or bone, provided they are perfectly sound. He strongly advocates this practice, for these advantages: Exarticulation is quicker, easier, requires simpler instruments, and is attended with far less bleeding than amputation in the continuity. The risks of septicæmia and osteomyelitis are reduced to the lowest attainable point, as the bone remains intact; less shock, less retraction of the muscles, less danger of injury to the flaps and nerves by the action of the unsawn bone; the power of sustaining the pressure of an artificial limb is earlier acquired and the point of support is broader and better fitted than when the bone has been divided.—*Braithwaite's Retrospect*, July, 1875.

2. *Nævi successfully treated with Electrolysis.*

J. B. Knott (St. Mary's Hospital, London) thinks this by far the best treatment, and gives a report of forty cases to corroborate his claims. He attaches* one or more needles to the negative and one needle to the positive pole of a continuous battery; they are passed into the nævus, and a short while afterwards bubbles of gas will be seen passing by the side of the needles, which indicates that decomposition begins to take place. The tumor turns into a firm clot of a bluish white color, and in this clot fibrous degeneration takes place. The number of sittings varies with the size of the nævus.—*London Lancet*, March, 1875.

3. *Scarless Eradication of Nævi.*

This is accomplished by a slow subcutaneous strangulation and ecrasing; and Richard Barwell (Charing-Cross Hospital, London) does it this way: He encloses subcutaneously the base of the tumor in a wire loop, both

* According to the size of the tumor.

ends of which emerge at the same opening in the skin. An oval rubber plate, about three-quarters of an inch long and one-eighth of an inch thick, has two holes bored obliquely through its thickness, and on its upper surface two little studs near the holes. The wire ends made to cross are passed through the holes in the rubber disk and drawn upon until the nævus is rather tense; then each end is twisted round a stud. When, on the third or fourth day, the wire becomes loosened, it is drawn tight again, and this process is repeated until the wire comes away. Thus the subcutaneous part of the nævus is destroyed and the cutaneous parts at once will shrivel and lose their morbid color. This change is hastened by the aid of strong nitric acid which is brushed over the colored parts, left for a few seconds and then washed off with an alkaline solution. —*London Lancet*, July, 1875.

4. *Varicose Veins.*

John Marshall (University College Hospital, London) removed nine inches of a badly varicose internal saphenous vein below the knee. He marked the course of the tortuous vein with ink. Esmarch's elastic bandage was then applied, and an incision was made through the skin from the upper to the lower end of the ink mark. The vein thus exposed was ligated at both ends and entirely removed with forceps and scissors. The operation was performed under the carbolized spray, and Lister's antiseptic method of dressing strictly employed. Patient was discharged from the hospital seven weeks after the operation.—*Braithwaite's Retrospect*, July, 1875.

5. *Treatment of Erysipelas by Hypodermic Injections of Carbolic Acid.*

Eugene Bockel gives (*Gazette Médicale de Strassbourg*) an account of a series of cases treated by the above method which, as far as we know, first was suggested by Prof. Hueter. Using an ordinary hypodermic syringe he makes in the circumference of the erysipelatous surface five to six injections, a syringefull each. These injections are repeated every morning and evening until the erysipelas is arrested in its progress. Sometimes this is accomplished by one series of injections ; generally, however, it takes three to four days.—*Memorabilien*, June, 1875.

### V. Practical Medicine and Pathology.

1. *Respiratory Percussion.*

Prof. DaCosta writes an article for the *Amer. Jour. of Med. Science* (July, 1875), under the above caption. Respiratory percussion is percussion with the patient holding his breath in full inspiration, or forced expiration. Under both of these conditions, the percussion note is found to differ from that elicited while the patient is breathing easily—that with full inspiration differing most, and this difference being most available in diagnosis. At the apices and infraclavicular region, a held inspiration makes the sound (in a healthy person) more resonant, fuller, and *the pitch higher ;* and where the left side has normally a higher pitch, this disparity is preserved. This statement holds good, in a greater or less degree, with the whole chest, although below the region mentioned it makes the normal disparity between the two sides less apparent.

A full and forced expiration lessens the resonance, and lowers the pitch over the entire lung on both sides—this being more clearly made out at the upper part of the chest anteriorly.

The increased resonance with raised pitch, he suggests may be explained by the fact of the altered tension of the structures in respiratory percussion.

Prof. D. has found this method of great value in diagnosis.

In *bronchitis*, where the secretions are very abundant, the breath sounds are often impaired, and the percussion note as well. The chest, struck while in full inspiration, gives exactly a normal sound. If, however, the disease has attacked the finer structures, and solidification is beginning, this manœuvre does not render the note fuller and more resonant. If the lung is merely collapsed, this method often gives an almost normal sound.

In acute lobar pneumonia, while nothing is learned during perfect hepatization, as resolution begins, the note heard on forced inspiration is more resonant and pulmonary.

Where, in a case of slight solidification, there is doubt whether it be caused by local congestion or tubercles, respiratory percussion solves the question—the case of local congestion becoming normally resonant, that of tubercles not.

In *pleurisy*, "over the seat of plastic exudation of ordinary extent," "forced inspiration diminishes the slight dulness that exists." Over the seat of marked effusion, no change is effected in the percussion note. In instances of pleuritic effusion, where there is blowing respiration at the back of the lung, and "the question arises whether or not pneumonia co-exists," respiratory percussion (inspiration) answers that question for us. Over the lower part of the chest, the note is unchanged ; so is it if the dulness be due to pulmonic solidification from inflammation ; but if it be due to condensation of lung tissue from compression, "decided clearness takes the place of dulness." In cases of tubercular deposit, at the early time when prolonged expiration just begins to be heard, and ordinary percussion detects little or no change, respiratory percussion makes the change more marked, and in this way : it develops a dulness previously not existing, or it makes the resonance of the damaged lung only slightly greater, and raises the pitch, "but does not bring out the changes strikingly, as in the healthy lung."

When ordinary percussion shows marked dulness, that on forced inspiration may show but little change, "except a slight rise in pitch, or the pulmonary resonance may be partly restored," and that in portions of lung still expansible and capable of function. Should in a given case this method fail, at a later examination, to show this restoration of resonance, or reveal it less than at a former exploration, it would be proof that real solidification had progressed.

Finally, in pulmonary emphysema, Prof. D. believes his discovery is infallible as a diagnostic indication. "In marked emphysema, the excessively clear or vesiculo-tympanitic note is unchanged by percussion during the act of breathing; when the emphysema is not so great, it is slightly changed."

2. *The Natural History of Acute Dysentery.* (*Amer. Jour. Med. Science*, July, 1875.)

Prof. Austin Flint reports ten cases of this disease in which medical treatment was omitted, for the purpose of studying the natural history of the affection. The cases were all of the sporadic variety, were hospital patients, and were carefully selected with a view to illustrating the whole course of the affection, with a diagnosis that was positive, and without medication, except *placeboic.* None of the ten cases ended fatally. The duration from the beginning of diarrhœa to convalescence varied from six to twenty-one days. The mean duration was eleven and one-fifth days. The results prove, as Prof. F. believes, that sporadic dysentery, "in a temperate climate, tends intrinsically to recover," and "that this disease has a self-limited duration." In 1853, Prof. Flint analyzed forty-nine cases of dysentery under treatment. Among these were thirteen hospital cases. The mean duration, in these thirteen cases, was just thirteen days. The mean duration of all that recovered (thirty) of the forty-nine cases, including, of course, those in private practice, was nine and five-sixth days.

Of the ten cases here reported, none of the patients had previously had dysentery, and in all the disease finished its course without any important complication, and it was not followed in any case by the chronic affection.

3. *On the Pathology and Treatment of Cholera.*

Surgeon A. R. Hall contributes to *The Practitioner* of July, 1875, a paper on this subject, which includes a paper, or the substance of it, read by him before the Royal Medical and Chirurgical Society in 1873. Surgeon Hall was for eleven years stationed in Bengal and had extensive observation of cholera, beside being at one time himself the subject of it. He strongly believes the true state of the sympathetic nerve in cholera is one of irritation and not of paralysis; that the morbid influence exercises a stimulating action on the vaso-motor nerves. Resulting from this there is increased heart's action, contraction of arterial walls, and at first augmented blood-pressure. At first the pulse is hard; afterward it becomes small from the small amount of blood in the artery. The copious limpid urine early is related to high arterial pressure; the total suppression later is due to cramp-like spasm of the renal arterioles.

The irritability of the sympathetic, through vaso-motor action, produces spasm of the walls of all the arteries. "The heart contracts forcibly but cannot dilate normally" on account of spasm—the inhibitory action of the vagus is defective. Post mortem—the right heart is gorged with blood, due to spasms of pulmonary arterioles; the left heart is empty from the same cause. The systemic veins are gorged with blood from arterial spasm. He suggests the cramps may be due to the cutting off of the blood supply by the contracted arterioles. The packed and drawn-back condition of the intestines is due to contraction of all their muscular fibres. The urinary bladder is found contracted to the "size of a walnut." Abortions occur in pregnant women attacked by cholera, the fœtus being born alive, which proves the accident to be due to spasmodic contraction of muscular fibres and not to death of the child. Everything points to extensive contraction of involuntary muscle. Logically, then, the best treatment is such as will counteract this condition.

"The hypodermic injection of pure sedatives" seems to accomplish this purpose. Chloral hydrate, by this mode of administration, is the best treatment for cholera collapse. Twenty cases are reported treated by this method, with only two deaths. The cases all occurred in India. A solution of chloral was made of 10 grs. to 100 minims of water, and 10 to 40 minims injected at a time—the intervals between doses being from half an hour to several hours, as the conditions seemed to demand. Within half an hour after the first injection the patients—in the most extreme state of collapse—uniformly expressed themselves as feeling "more life."

4. *Incarceration of Jejunum and Intussusception.*

In the *Med. and Surg. Reporter*, July 10, 1875, Dr. A. R. Kilpatrick, of Texas, records a case of incarceration of jejunum and two cases of intussusception. The first was in a negro woman of 40, very fat, who had at her confinement, four months before, received some internal injury, as was thought, which was followed by fever and severe pain in the region of the umbilicus. She frequently thereafter felt pain in this region, and on exposure to dampness, and probable cold-catching, she had here violent pain and a considerable swelling. She soon had symptoms of peritonitis and enteritis; all cathartics failed to act, and stercoraceous vomiting came on. She died in four days. Post mortem, it was found that about four inches of jejunum had become incarcerated between two bridles made by adhesion of the omentum to the mural peritoneum, and about two inches apart. By absorption of fat, a pocket had been formed in the very thick abdominal wall for the incarcerated intestine, so that the latter approached to within a fourth of an inch of the cutaneous surface. This explained the prominent tumor near the surface, and suggested that probably the adhesions occurred at the time of confinement referred to.

The cases of intussusception, curiously, occurred in a mother and child at the same time. They were negroes, and, respectively, 36 and 13 years old. Both were attacked with fever, went to bed and were given rhubarb and "other things." Both purged violently, had pain with the purging, and finally vomiting came on, which continued in spite of all attempts to arrest it. Both died at the end of three or four days.

Post mortem, in the woman, the small intestines were found invaginated in seven different places, "the extent of each place varying from three to fourteen inches. Some had evidently been but recently ensnared, while others were gangrenous." In the boy, the small intestine was found invaginated in five places and to various lengths. There was much inflammatory exudation. The patients, it was afterwards learned, were unusually susceptible to the influence of rhubarb; a very small portion of the drug purging them severely.

[In view of the very similar predispositions these patients (the last two) must, from their consanguinity, have had; of the apparent full action of the purgative and of their great susceptibility to the effects of the particular drug; of the statement that the vomiting only came on "finally," meaning probably some time after the purgative, and remembering the belief now held by eminent physicians that invagination in healthy people is not unusual, always correcting itself, the query seems natural whether, in the cases here reported, the invaginations were not produced by the purgative, and prevented from self-adjustment by some inflammation the medicine may have lighted up.—Eds.]

### VI. Therapeutics.

1. *Therapeutics of Trismus Nascentium.*

The exhibition of remedies in any disease is almost wholly controlled by our knowledge of the pathological character of that disease. In no malady has there been a greater amount of dispute about its pathology than in tetanus nascentium. In 1848, Dr. Marion Sims, then living in Montgomery, Ala., promulgated a theory of the pathology of trismus, which at that time produced a profound impression upon the profession in the United States and Europe. He contended that the cause of this morbid condition was "*centric*," owing to pressure on the medulla, by the occipital bone being depressed, in the process of parturition. This theory seemed so correct that it captivated many practitioners at first. Many a case was treated successfully immediately thereafter in accordance with this pathological idea, by what may be called, the *postural* treatment. But very many other cases were not successfully treated by this means, and thereby many were led to believe that Dr. Sims' ideas were not correct. This want of credence has so gained ground that, at the present day, a few books on the diseases of children mention this theory, under the head "Pathology of Trismus Nascentium," as one of the very many useless theories advanced to explain the cause of this malady. Dr. Sims' article is remembered by most of the senior members of the profession of to-day, but his treatment is seldom resorted to at present. The successful management of a case of tetanus infans, at present, is regarded as a most happy issue from an almost invariably—so considered—fatal disease. Warm baths, ice to the spine, hot water to the spine, blisters, mustard, narcotics, anæsthetics, amyl, and a host of other remedies, have been recommended and used with varying results; *so* varying, that to this day no one course of treatment may be said to be *successful.*

In the June number of the *American Journal of Medical Sciences*, is an article on the treatment of this distressing affection, from the pen of Philip A. Wilhite, of Anderson C. H., S. C., advocating Dr. Sims' pathology and treatment.

The writer resorts to the *postural* treatment. Babies, in trismus are almost invariably found lying on the back

—a position favoring the condition of depressed occipital bone. The usual concomitants are present in all degrees of intensity, from *mild trismus* — "*trismoid*" as Dr. Sims calls it — to the terribly destructive form, which kills the infant in one hour. The writer enumerates a number of cases in which he used postural treatment with charming success. The postural treatment consists in placing the infant on its side. In this position the pressure is removed from the brain *at once* in most cases, and amelioration is marvelous. The little patient usually sinks into a quiet slumber, and when awakened it takes the breast promptly and without any hesitancy, showing that the condition of locked jaw is removed. One case is here quoted from: A woman had triplets; two had died of trismus within a few hours of each other. The third was writhing in the *last* agonies, as the friends supposed, of this malady, when the doctor, casually passing the house, stepped in and was shocked to find two dead babies in the coffin, laid aside, awaiting the death of the third baby, so that all three could be interred together. "Notwithstanding the fact that the child seemed almost moribund, I stated to the parents and friends that I wished to try the experiment of the lateral decubitus. They at first objected to my doing anything at all, saying the child must die as the others had, and that it would soon be out of its misery. Mrs. R.—the mother—said it had not been able to swallow for the last fifteen hours, and what was the use of worrying a dying child? However, I carried my point and had the child laid lengthwise on a soft pillow on its side, and turned from side to side every fifteen minutes. From the moment it was laid on the side, a decided change was seen in all its symptoms, and in less than an hour and a half from the time the lateral decubitus was instituted, all spasm ceased, the jaws were unlocked and the child sucked freely and swallowed well. I gave it no medicine, but simply directed that lateral position should be kept up for some time, being careful to change it from side to side every hour or so. * * * * It recovered rapidly and I saw it no more till it was eight months old; and there was not a finer child in the country, of its age."

Examination of the two dead babies revealed "the occiput, in each, depressed to an unusual degree."

The pathology of this malady suggests the treatment, viz., removal of pressure by the occiput. To accomplish this, placing the infant on one side and then on the other, alternately, changing its position every fifteen or thirty minutes, will generally suffice, as will be evidenced by a diminution or a cessation of the convulsions. When this does not relieve the patient, surgical interference should be promptly resorted to, to elevate the bone.

